# Early contribution of germline and nevi genetic alterations to a rapidly-progressing cutaneous melanoma patient: a case report

**DOI:** 10.1186/s12920-022-01426-2

**Published:** 2023-01-05

**Authors:** Ana Mordoh, Juan Carlos Triviño Pardo, Ibel Carri, María Marcela Barrio, José Mordoh, Mariana Aris

**Affiliations:** 1 Hospital de Clínicas, Buenos Aires, Argentina; 2 Sistemas Genómicos Grupo Biomédico ASCIRES, Valencia, Spain; 3 Instituto de Investigaciones Biotecnológicas, Universidad Nacional de San Martín, Buenos Aires, Argentina; 4 Centro de Investigaciones Oncológicas-Fundación Cáncer, Buenos Aires, Argentina; 5 Fundación Instituto Leloir, IIBBA-CONICET, Buenos Aires, Argentina; 6 Instituto Alexander Fleming, Buenos Aires, Argentina

**Keywords:** Case report, Cutaneous melanoma, Tumor progression, Whole-exome sequencing, Germline alterations, Somatic alterations, Cancer pathways, Cancer hallmarks, Nevi heterogeneity

## Abstract

**Background:**

Cutaneous melanoma is the skin cancer with the highest mutational burden and metastatic rate. Early genetic alterations and biomarkers of distant progression are a point of interest. In addition to germline-susceptibility loci, almost 30% of melanomas arise from precursor benign nevi lesions, providing a source for malignant transformation.

**Case presentation:**

Patient#009 developed a cutaneous melanoma over a nevus, followed by progression to regional and distant metastases in months, unresponsive to targeted therapy. To search for the genetic contribution to this rapid progression, a longitudinal analysis was performed through WES of germline, nevi, primary tumor, and a metastatic lymph node. Differential SNP/INDEL and CNV gene alterations, with functional impact on key pathways and cancer hallmarks in each step of evolution, were discerned. Tumor-associated nevus was, for the first time, split into two sections, distant and adjacent to the primary tumor, to study its heterogeneity.

Shared SNP alterations, with stable allele fraction from germline to metastasis were detected, mainly affecting DNA repair genes and promoting genome instability. Early somatic alterations, shared by nevi and primary and metastatic tumors, included BRAF^V600E^ and focal copy-loss of several genes, acquiring additional cancer hallmarks. Phylogenetic analyses revealed that these common somatic alterations would provide a “bridge”, allowing progression from a benign to a malignant state. Distant and adjacent nevi were rich in alterations, presenting differential SNP and CNV alterations. Upon tumor transformation, a marked increase in CNV over SNP alterations was determined. Both the number of SNP and CNV-affected genes, including known driver genes, increased throughout progression, although TMB levels remained lower than expected for melanoma. Typical alterations in BRAF^V600E^ tumors related to intrinsic resistance to targeted therapy were found, including BRAF amplification and loss of PTEN, CDKN2A/B, and TP53 surveillance genes. Finally, numerous metastatic alterations were detected, further promoting tumor progression.

**Conclusions:**

In this patient, longitudinal WES analysis revealed a sequential and cumulative pattern of genetic alterations, where germline and nevi somatic events contributed early to its rapid clinical progression. In this case report, we found tumor-associated nevi as genetically heterogeneous precursor entities, in which potential prognostic biomarkers should be studied prospectively.

**Supplementary Information:**

The online version contains supplementary material available at 10.1186/s12920-022-01426-2.

## Background

Cutaneous Melanoma (CM) is the most aggressive skin cancer, with the highest mutational burden and metastatic rate [1, 2]. Short Single Nucleotide Polymorphisms and Insertions–Deletions (SNP/INDEL) dominate the biology of CM, in contrast to acral/mucosal melanomas, where large structural variants prevail [3]. Early genetic alterations fostering distant progression are a point of interest. According to The Cancer Genome Atlas (TCGA), around 8% of adult cancers have pathogenic germline variants, including 6% of CM cases; most predisposition genes affect genome integrity, belonging to the core of DNA-damage response genes [4]. In CM, a meta-analysis of Genome-Wide Association Studies (GWAS) from case–control studies found 20 melanoma-susceptibility loci, with high-to-low penetrance variants reaching genome-wide significance, including DNA repair genes such as PARP1 and ATM [5]. Defective DNA repair in melanocytes may play a dual role, allowing nevus formation as well as the accumulation of UV-induced mutations [6].

Although the malignant transformation of nevi is a rare event, the number of nevi is considered a risk factor for developing melanoma; approximately 30% of CM arise within or adjacent to a pre-existing nevus [6–8]. Previous genomic analyses confirmed the clonal origin of melanomas from adjacent nevi [9]. In these studies, nevi had been taken as a whole for molecular analysis. Prior reports established that nevi were associated with exclusive driver mutations in the MAPK-pathway, typically NRAS^Q61R/L^ for congenital nevi and BRAF^V600E^ for acquired nevi; although cross-mutations were also reported [10]. Deleterious mutations in tumor suppressor genes are consistently absent in nevi, despite being a trait for melanoma formation. Regarding gene Copy Number Variation (CNV) in nevi, there are conflicting reports in the literature. The first studies, performed by Bastian et al*.* by microdissection and targeted sequencing, found no copy-genomic alterations on CM-associated nevi (*n* = 37) [9]; subsequent extended analyses revealed no differences [11]. WES analysis performed on nevi by Stark et al*.* distinguished extensive CNV-wide losses in reticular acquired nevi, balancing the number of affected tumor suppressor genes and oncogenes, resulting in a neutral effect [6]. However, in an extended cohort of 8 nevi, minimal CNV regions were found (range: 2–29) [12]. Lozada et al*.* analyzed 12 melanocytic nevi by targeted sequencing, reporting few CNV alterations [13]. In this study, a CM-associated nevus lacked high levels of amplifications and deletions, exhibiting common focal CNV regions among in situ and invasive melanoma, which are a hallmark of CM.

In this case report, we describe the genetic landscape of a CM patient with rapid clinical evolution through longitudinal WES analysis of germline, precursor nevi, primary tumor and metastasis, characterizing the main alterations acquired at each step of tumor formation and progression. To determine whether nevi are heterogeneous entities, we performed genomic analysis of two well-differentiated nevi sections: distant and adjacent to the primary CM. In this way, we aimed to confirm and identify differential alterations in the precursor nevus, depending on its proximity to the primary tumor, which had formed on it.

## Case presentation

**Fig. 1:**
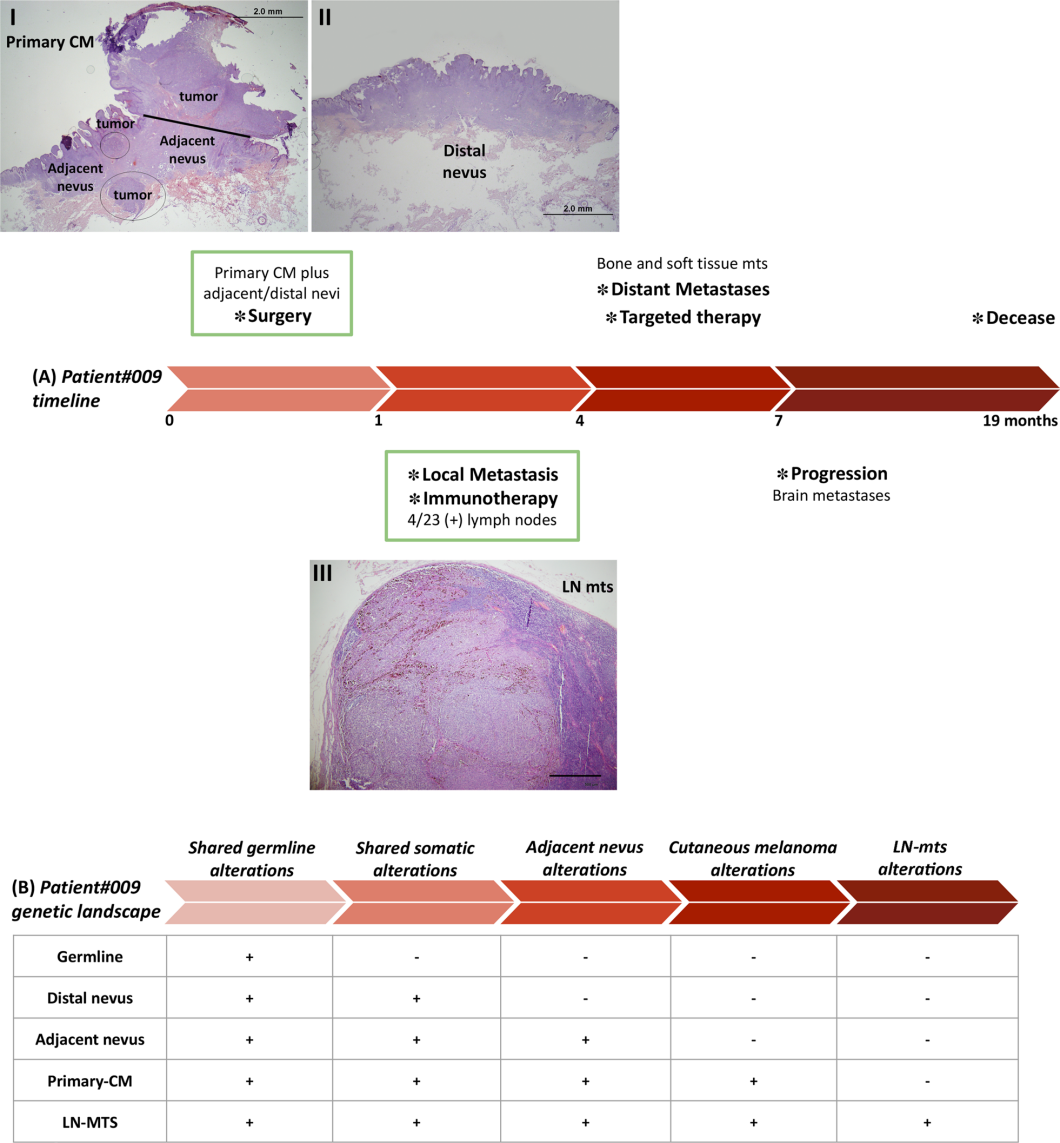
Patient#009 timeline and genetic landscape. **A** Clinical evolution of patient#009 including the main events. Biopsies analyzed in this case report are indicated with a green box, and representative HE-histology images at low magnification are shown: (I) adjacent nevus and primary-CM (2 mm), distal nevus (2 mm), and LN-mts (500 µm). **B** Longitudinal WES analysis of patient#009 throughout cancer progression, with differential SNP/CNV alterations at each step of clinical evolution: *shared germline alterations*, including germline alterations present in all tissue samples; *shared somatic alterations*, including somatic alterations common to all tissue samples; *adjacent nevus alterations*, including alterations present in the adjacent nevus and not in the distal nevus; *melanoma alterations*, including common tumor alterations to primary-CM and LN-mts; and *LN-mts alterations*, including only alterations proper of the metastasis. All analyses performed are fully described under Methods **(**Additional file 1)

The clinical evolution of this patient was previously described [14]. Briefly, a 45-year-old Caucasian woman (patient#009), with neither a family background nor a history of skin chronically exposed to sun, underwent a large surgery resection of a spontaneously bleeding tumor in her back (Fig. 1A). The histopathological biopsy analysis after surgery revealed an ulcerated nodular primary-CM associated with a nevus, with a Breslow thickness of 7.8 mm, and an MKI67 proliferative index (PI) over 30% (Fig. 1A, I). For histological considerations and genomic analysis, the nevus was split into 2 sections: adjacent to the primary-CM (*adjacent nevus*) (Fig. 1A, I); and distant from the primary CM (*distal nevus*) (Fig. 1A, II). Both nevi had a PI of 2%. A month later, 4/23 positive metastatic lymph nodes were detected in her right axilla; a representative lymph node metastasis (LN-mts) with a PI over 30% was analyzed in detail (Fig. 1A, III). Patient#009 was diagnosed as CM stage IIIC, and following the selection process to enter the CASVAC-0401 trial [15], she was randomized to receive the allogeneic cellular vaccine VACCIMEL. However, a month later, after receiving only one vaccine dose, patient#009 started with lumbar and rib pain. A PET scan revealed bone metastases at the 9th-left rib and the right-acetabulum, and soft-tissue metastases at the right infra-axillary region. Therefore, this patient was at stage IV (M1c) and had to discontinue the CASVAC-0401 protocol. Bone metastases were irradiated and, as tumors were BRAF^V600E^ positive, Vemurafenib was administered (960 mg orally twice a day). However, patient#009 further progressed developing brain metastases only 7 months after CM diagnosis (stage IV, M1d), and passing away 12 months later (Fig. 1A). The rapid evolution of this case led us to wonder which early genetic alterations fostered transformation, including germline and different nevi sections; and which later alterations promoted tumor progression, including primary-CM and an accessible regional LN metastasis. Therefore, the presence of gDNA alterations in genes, either SNP/INDEL or CNV, was determined in each sample by whole-exome sequencing (WES) (Additional files: 1, 2, 3 and 4). An overall picture of the genomic alterations in patient#009 revealed that CNV prevailed over SNP/INDEL; gene deletion was more frequent than amplification **(**Additional file 5**)**. Analysis of the tumor mutational burden (TMB) revealed low although increasing levels of SNP per Mb throughout tissue samples (Additional file 5A). Also, the proportion of genome regions affected by CNV increased upon malignant transformation per tissue sample (Additional file 5B). Ploidy estimation did not detect any genomic duplication in samples A-D (2–2-2.65–2.55). Phylogenetic analysis revealed a division between nevi and tumor samples, with LN-mts detaching from primary-CM, and a slight separation between distant and adjacent nevi (Additional file 5C).

**Fig. 2:**
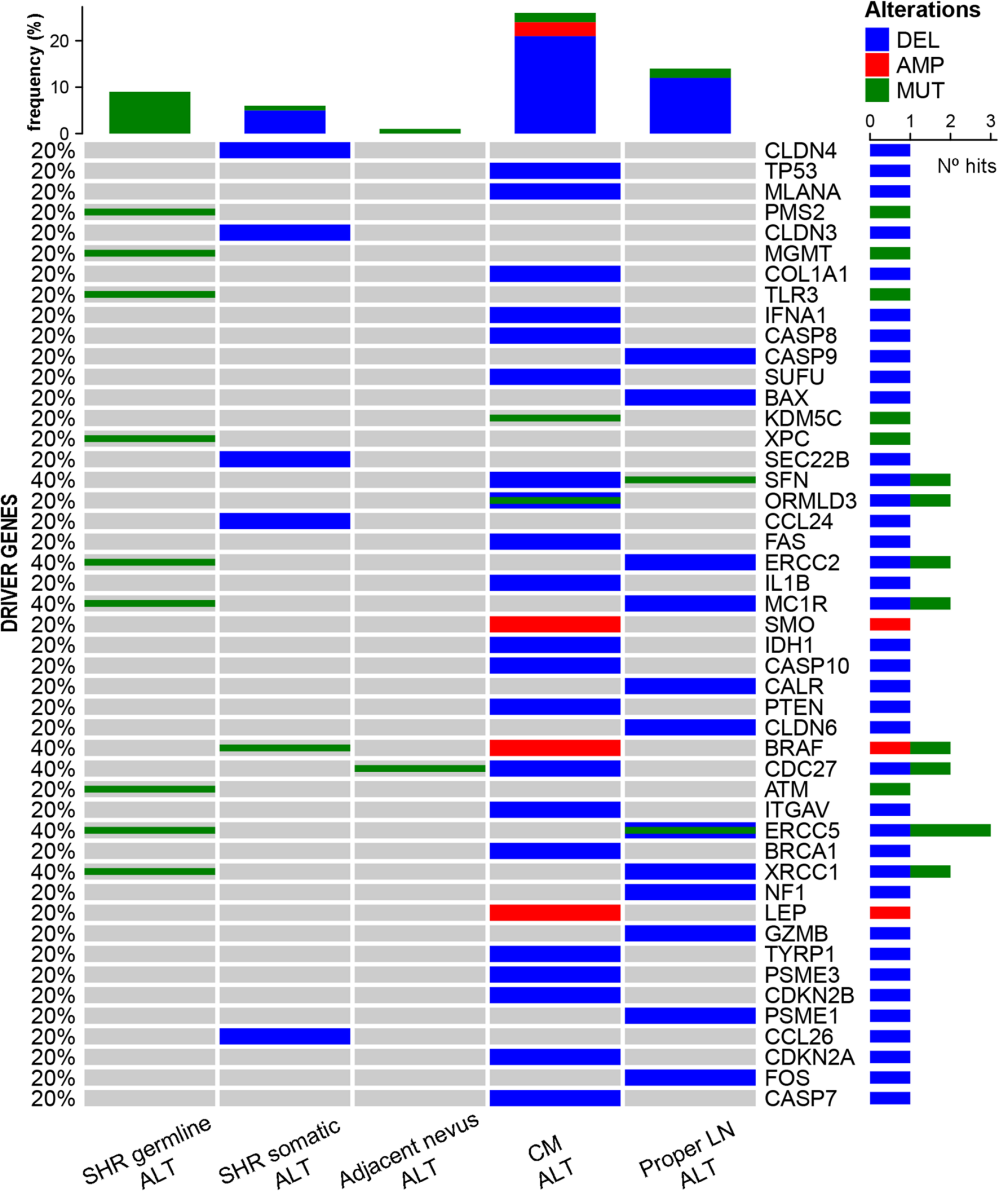
Cancer driver genes throughout the progression of patient#009. Oncoprint representation of selected driver genes throughout the progression of patient#009 are shown, indicating SNP/INDEL alterations in green, CNV amplifications in red and CNV deletions in blue. In the right axis, the total number of hits or alterations on each gene throughout progression is shown. *Subsets:* shared germline alterations (SHR germline ALT), shared somatic alterations (SHR somatic ALT), adjacent nevus alterations, CM alterations, LN-mts alterations

**Fig. 3:**
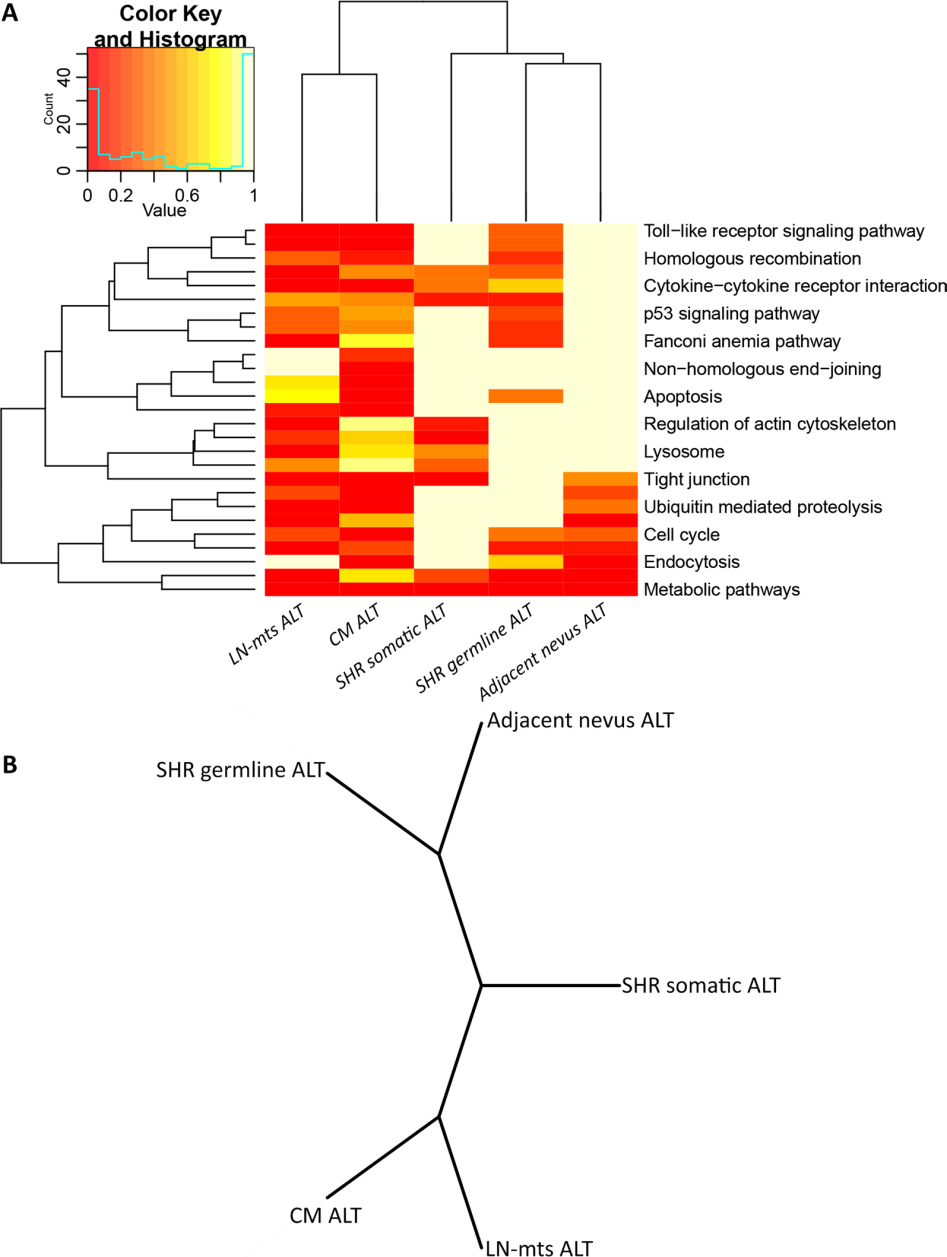
Key pathways throughout the progression of patient#009. **A** Heatmap representing hierarchical clustering of each step of progression (columns) and KEGG cancer-related pathways (rows), analyzed by functional enrichment. The color key and histogram related to *p*-values are indicated in the upper box. A *p*-value adjusted by FDR of 0.05 has been selected as a significant threshold. **B** Tree visualization of phylogenetic distances between each step of progression, according to their SNP and CNV alterations. *Subsets:* shared germline alterations (SHR germline ALT), shared somatic alterations (SHR somatic ALT), adjacent nevus alterations, CM alterations, LN-mts alterations. All analyses performed are fully described under Methods (Additional file 1)

To perform a functional analysis, we focused on the gain/loss of function in critical genes and pathways related to cancer, in each step of tumor transformation and progression (Fig. 1B). We looked forward to analyzing whether there were any germline alterations common to all tissue samples, related to melanoma susceptibility and poor prognosis; whether there were any somatic alterations shared by nevi and tumor biopsies; what differences, if any, were there between distant and adjacent nevi; and which alterations fostered malignant transformation and metastasis. To begin with, we filtered a list of cancer driver genes from COSMIC in our data, finding several affected genes in the germline, which increased throughout progression; a panel with key-selected driver genes is shown in an oncoprint (Fig. 2; Additional file 4). In addition, we filtered a list of genes associated with KEGG pathways related to cancer [16] in our data for enrichment analysis, in search of statistically-significant enriched pathways in each step of patient#009 evolution (Fig. 3A, Additional file 4).

Shared germline alterations (Fig. 1B) showed a similar bimodal allele-frequency distribution, typical of heterozygous and homozygous variants, with a stable allele fraction from germline to LN-mts (one-way ANOVA test, *p* > 0.05) (Additional file 6A). Among the 60 SNP detected in melanoma-susceptibility genes, only those loss-of-function alterations were analyzed (*n* = 12) (Additional files 2, 4). Analysis through the KEGG resource revealed a functional enrichment in the *nucleotide excision repair* (NER) pathway, affecting ERCC2, ERCC5, and XPC genes; other DNA repair pathways also showed affected genes, early affecting genome stability (Fig. 2, Fig. 3A). Functional enrichment through GO and Reactome resources was also related to terms and pathways relative to DNA repair (Additional file 7).

Regarding shared somatic alterations **(**Fig. 1B), the main alteration was the early gain-of-function of the BRAF^V600E^ oncogene from nevi to tumors (Fig. 2, Additional file 4). Few SNP were detected, complemented by copy-number loss of genes with increasing cellular fraction in LN-mts (Additional file 6B, C). KEGG enrichment analysis revealed that *tight junctions* and *regulation of actin cytoskeleton* pathways were affected, promoting invasion and metastasis; and *SNARE interactions in vesicular transport* were also affected, evading immune destruction (Fig. 3A, Additional file 4). The comparison of adjacent with distant nevi relative to the primary CM (Fig. 1B) revealed differential SNP and CNV alterations detected only in the adjacent nevus; these alterations decreased their frequency towards LN-mts, while proper adjacent nevus alterations occurred at low frequency (Additional file 6D, E, F). These differential alterations were enriched in genes from *base excision repair* (BER), *endocytosis* and *proteasome* pathways (Fig. 3A, Additional file 4).

Upon malignant transformation (Fig. 1B), an increasing number of SNP and numerous copy-number gains and losses on genes were detected. Common alterations to primary-CM and LN-mts exhibited higher frequency in the latter; the few proper alterations of the primary-CM were of low frequency (Additional file: 6G, H, I). The main events included the copy-number gain of BRAF^V600E^, further contributing to the activation of the MAPK pathway. Other events included evading growth suppressors through loss of CDKN2A, CDKN2B, PTEN and TP53 from the *cell cycle* pathway; resisting cell death through loss of CASP7, CASP8, CASP10 and FAS from the *apoptosis* pathway; and genome instability through loss of genes from the *non-homologous end-joining* (NHEJ) pathway (Fig. 2, Fig. 3A, Additional file 4). In addition, the loss of several chemokines, interleukin and interferon genes involved in *cytokine-cytokine receptor interaction* and *toll-like receptor-signaling* pathways, along with *antigen processing and presentation* pathways, contributed to evading immune destruction. Other enriched pathways included *ECM-receptor interactions, tight junctions*, and *endocytosis* (Fig. 3A). Functional enrichment through GO and Reactome resources revealed similarly affected terms and pathways (Additional file 7). Interestingly, some genes exhibited double events (SNP plus copy-deletion), such as CDC27 and SFN from the *cell cycle* pathway, and ORMLD3, a negative regulator of sphingolipid synthesis (Fig. 2).

Finally, proper alterations of LN-mts (Fig. 1B), which were numerous and of low allele fraction, included SNP and copy-number loss; no amplified regions were detected (Additional file 6J, K). These contributed to genome stability by affecting more genes from *BER, NER, Fanconi anemia (FA), and melanogenesis* pathways (Fig. 3A, Additional file 4). In addition to germline mutations, MC1R, XRCC1 and ERCC2 genes lost a copy in LN-mts; and ERCC5 displayed a second-somatic SNP in LN-mts, adding to its first hit in the germline (Fig. 2). Also, more enriched KEGG pathways affected genes from *cytokine-cytokine receptor interaction* *toll-like receptor-signaling*, *regulation of actin cytoskeleton, tight junctions, lysosome, proteasome,* and *ubiquitin-mediated proteolysis* pathways (Fig. 3A, Additional file 4). These results were also reflected in GO and Reactome resources (Additional file 7).

Interestingly, when evaluating phylogenetic distances among all these altered genes from the different steps of transformation and progression, we observed a clear separation of pre-malignant and malignant alterations; while we found shared somatic alterations as a nexus between pre-tumor alterations (germline/nevus) and tumor-associated alterations (primary-CM/LN-mts) (Fig. 3B). When analyzing the KEGG-enriched pathways, we noted that this clear separation between benign and malignant alterations was conserved, with somatic alterations in common between them (Fig. 3A).

## Discussion and conclusions

In this case report, the genetic evolution of a CM patient contributing to her rapid clinical progression was analyzed through a longitudinal WES analysis. The possibility of evaluating whether alterations would impact driver genes sequentially, in specific steps that underlie tumor progression, ideally requires the evaluation of precursor lesions, located next to the tumor. Our goal was to discern the differential alterations with an impact on the key pathways that have occurred in each step of tumor transformation and progression, rather than a description of alterations in individual samples.

Although this case did not fulfill the clinical criteria of familial melanoma, numerous variants previously described as related to susceptibility and poor prognosis in CM were detected, including germline SNP in ERCC2, ERCC5 and XRCC1 [5, 17–19] [4, 20]. This early alterations affected the maintenance of genome integrity; with further loss of function mainly through somatic copy-deletion of additional DNA repair genes along with progression, and a double-hit in ERCC5. Recently, pathogenic germline variants were reported as the highest-risk biomarker associated with poor survival in stage III/IV melanoma patients [21]. Melanogenesis was also early-affected, with MC1R having a germline SNP followed by gene-loss in LN-mts, plus copy-loss of several genes upon transformation typical of differentiated melanocytes, such as TYRP1 and MLANA. Beyond its role in inducing pigmentation, there is increasing evidence that MC1R promotes the repair of UV-induced DNA damage; therefore, its loss presumably promoted genome instability [22].

BRAF^V600E^ was the leading alteration common to all biopsies, found in both nevi sections and tumor tissues. In addition, focal CNV regions, which are a hallmark of CM, were found both in nevi and tumor samples, affecting the copy-loss of genes involved in tight junctions (CLDN3, CLDN4, TJP1 and TUBA3D, TUBA3E), and vesicular-transport molecules (SEC22B, STX1A). These early somatic alterations indicate that nevi would need additional driver alterations other than BRAF^V600E^ to create the right background for the subsequent development of aggressive melanoma. To determine whether nevi are heterogeneous entities, as they tend to be considered a whole entity, we performed WES analysis on two nevi sections: distant and adjacent to the primary-CM. To our knowledge, this is the first time that this kind of analysis has been performed; revealing differential alterations between them. Mainly, these differential alterations were enriched in DNA repair, endocytosis and proteasome pathways. Some alterations were common to the adjacent nevi, primary-CM and LN-mts, suggesting that the adjacent nevus is an intermediate lesion between the distant nevus and the primary-CM. It is interesting to note that genetic abnormalities in both nevi sections were not reflected in their PI, both having 2% MKI67^+^ cells; while upon transformation, PI increased by over 30%. As mentioned in the introduction, there are conflicting reports about genetic alterations in nevi; most of them described only the key-driver SNP in BRAF and NRAS oncogenes. The extension of CNV-affected regions is variable, and no functional analyses of the CNV-affected genes were previously described. In this patient, we found SNP as well as CNV-affected genes common to nevus and tumor lesions, supporting their clonal relatedness. Through bioinformatics functional analyses, we found that these early-somatic deleted genes might have promoted several cancer pathways. Interestingly, phylogenetic analyses revealed that these shared somatic alterations, detected in distant and adjacent nevi and remaining in primary-CM and LN-mts, would provide a “bridge”, allowing progression from a benign to a malignant state. These results support the idea of precursors nevi being genetically heterogeneous and rich in genetic alterations. Persistence over time of these early alterations in tumor tissue would support their role in CM transformation and progression, suggesting that the fate of CM proceeding from nevi can already be defined, at least partially, in the precursor lesion.

The genetic landscape of this CM-patient revealed a sequential and cumulative evolution of genetic abnormalities, which began early with germline SNP common to all samples, followed by somatic SNP and CNV alterations, found in the precursor nevi and common to all tissues. In this case, upon transformation, the number of genes affected by SNP and CNV alterations increased throughout progression, with a prevalence of gene copy-number loss. A priori, the TMB in tissue samples revealed lower values than expected for most CM tumors. However, along with germline alterations, low TMB and BRAF^V600E^ were reported as poor progression-free survival biomarkers for CM patients [21]; this case combines all these somber factors. Presumably, a low TMB is related to a low number of tumor neoantigens, and therefore to a poorer anti-tumor immune response. Instead, this case revealed a marked increase in the number and extension of CNV regions with progression. Altered genes in the germline involved most DNA repair pathways, including HR, NHEJ, and FA, which might have promoted early double-strand breaks and chromosome instability. Some alterations typical of BRAF-mutated melanoma tumors were detected in this patient [1], such as BRAF amplification, loss of NF1 and PTEN, loss of CDKN2A/B and TP53 surveillance genes, and mutation of KDM5C epigenetic regulator. Nevi senescence might have been overcome early through germline SNP in ATM, followed by CDKN2A and PTEN loss, and EZH2 amplification; as all these genes contribute to cell-cycle arrest [23–26]. The main affected pathways reported for patient#009 are in line with a previous landscape study in 82 melanoma patients, showing gradual accumulation of SNP and CNV alterations activating MAPK pathway, modulating the chromatin landscape, promoting cell cycle, disrupting the p53 pathway, and activating the PI3K pathway [11]. Interestingly, these typical alterations support intrinsic mechanisms for vemurafenib resistance, providing evidence of the rapid clinical evolution observed in this patient [27, 28]. Also, the loss of melanocyte-antigens such as MLANA has been associated with a phenotypic switching towards dedifferentiation to an invasive phenotype [29].

In this case report, a longitudinal WES analysis, from germline to benign and tumor lesions revealed a sequential and cumulative pattern of genetic alterations affecting key cancer pathways, where germline and nevus somatic events contributed early to its rapid clinical progression. A tumor-associated nevus was split for the first time into two sections for WES analysis, distant and adjacent to the primary tumor, revealing nevi as genetically heterogeneous precursor entities. It will be most interesting to analyze more cases with high-risk CM-associated nevi to further search for potential biomarkers of tumor transformation and progression with an impact on clinical practice, including the affected genes and pathways found in the present work.

## Supplementary Information

**Additional file 1.** Extended Materials and Methods.

**Additional file 2.** SNP/INDEL position and allele fraction per sample.

**Additional file 3.** CNV regions and cellular fraction per sample.

**Additional file 4.** Functional analyses of key-selected genes throughout progression.

**Additional file 5.** Characterization of somatic alterations from patient#009.

**Additional file 6.** Frequency distribution of alterations in patient#009 throughout progression.

**Additional file 7.** Complementary functional analysis of altered genes in Patient#009.

## Data Availability

The datasets generated and/or analyzed during the current study are available in the European Genome Phenome Archive (EGA), accession number EGAS00001006459.

## Abbreviations

BER: Base excision repair
CM: Cutaneous melanoma
SNP/INDEL: Single nucleotide polymorphism/Insertion–deletion
CNV: Copy number variations
FA: Fanconi anemia
HR: Homologous recombination
LN-mts: Lymph-node metastases
NER: Nucleotide excision repair
MMR: Mismatch repair
NHEJ: Non-homologous end-joining
PI: Proliferative index
TMB: Tumor mutational burden
