# Supplementary material for: Early contribution of germline and nevi genetic alterations to a rapidly-progressing cutaneous melanoma patient: a case report
Source: BMC Med Genomics. 2023 Jan 5;16:1. doi: 10.1186/s12920-022-01426-2 (PMC9814418; doi:10.1186/s12920-022-01426-2)
Supplement: Supplementary file 1 — Additional file 1. Extended Materials and Methods. [file 12920_2022_1426_MOESM1_ESM.pdf]

ADDITIONAL FILE 1: EXTENDED MATERIALS AND METHODS

Histopathological and immunohistochemical analysis of tumor biopsies

Tissue biopsies: primary-CM; distal nevus from primary-CM; adjacent nevus from primary-CM; and a Lymph-Node Metastasis (LN-mts). Timeline indicating the evolution of patient#009 and resection of tissue biopsies are shown in **Figure 1A**. Sections were examined by optical microscopy (Olympus BX40 microscope, DP2-BSW software), and digitized pictures were analyzed with ImageJ software (NIH). Histopathological features were determined on hematoxylin & eosin (HE) stained slides by our Pathology Department, according to AJCC-UICC staging [1]. Proliferative index (PI) was determined by MKI67 staining (PI) (%)= $MKI67^{+}$tumor cells/($MKI67^{+}$ tumor cells + $MKI67^{-}$ tumor cells) x100 [2]. PI was determined in 1 mm^2 of a tumor hot-spot zone. Immunohistochemistry was performed on tissue slides using anti-MKI67 mAb (clone MIB-1, Dako), and revealed with the Avidin-Biotin-Peroxidase (ABC) system and DAB (3,3′-Diaminobenzidine) (Vectastain, Vector Labs). $BRAF^{V600E}$ status of patient#009 was previously determined in tumor tissues by Sanger sequencing [3].

Samples and Whole-Exome Sequencing

All areas from FFPE tissues were enriched by laser microdissection (Leica DM/LAM). gDNA was isolated with RecoverAll (Ambion). gDNA from PBMC was used as the germline reference. DNA concentration and quality of all samples were verified by spectrophotometry (2000 Nanodrop, ThermoFisher Scientific) and capillary electrophoresis (2100 Agilent Bioanalyser, Agilent Genomics). Whole-exome sequencing (WES) was performed from the gDNA extracted from the different tissue samples and the germline reference, processed with the Agilent SureSelect All Exon Human V5 Library in an Illumina Hiseq 4000 PE101 platform (BGI, Hong Kong, China) that captured a 50.38 Mb target region. Total clean reads per sample were aligned to the human reference genome (GRCh37/HG19) using Burrows-Wheeler Aligner (BWA) [4]. Duplicate reads were eliminated with Picard tools (v2.5). On average, 99.63% mapped successfully, duplicate reads were removed. Of total effective bases, 50.64% were mapped on target regions. The mean sequencing depth on target regions was 102-fold. On average per sequencing sample, 99.83% of targeted bases were covered by at least 1X coverage and 98.75% of the targeted bases had at least 10x coverage. WES data from patient#009 samples were uploaded to the European Genome Phenome Archive (EGA); the corresponding accession number is EGAS00001006459.

DNA sequence data analysis

Variant calling was performed following Genome Analysis Toolkit (GATK) best practices [5]. To detect DNA alterations present in the germline sample, genomic variations, including single-nucleotide polymorphisms (SNP) and insertions/deletions (INDEL) were detected with HaplotypeCaller (v3.3.0) of GATK. Afterwards, the hard-filtering method was applied to get high-confident variant calls; which were annotated with the SnpEff tool (http://snpeff.sourceforge.net). Detection of melanoma susceptibility and negative-prognostic variants in the germline was performed by comparison to available databases and previous reports [6–9]. Only those susceptibility/prognostic variants present in the germline and all tissue samples from patient#009 were considered for analysis (*common germline alterations*). Allele fraction (AF) was estimated for each variant in all samples from the proportion of the sample's reads that support the variant allele(s), allowing state hetero/homozygosis and eventual changes with tumor progression.

To identify somatic SNP and INDEL, Mutect2 from GATK version 3.8-0 was applied using COSMIC version 82, dbSNP build 138, removing soft-clipped bases. PASS variants from Mutect2 with minimal coverage of five reads were analyzed **(Additional file 2).** Selected variants were annotated with CRAVAT [10] and Variant Effect Predictor (VEP, Ensemble) [11] resources. Tumor mutational burden (TMB) *per tissue sample* was calculated as the total number of somatic SNP plus INDEL mutations per exome size (50.38 Mbp), giving the number of SNP/INDEL per a megabase (MB) of sequenced genome (TMB) (**Additional file 5A**).

To determine Copy Number Variation (CNV), the algorithm FACETS v0.6.0. was applied with default parameters [12]. FACETS is an open-source integrated stand-alone pipeline applicable to NGS platforms involving sequencing BAM file post-processing, joint segmentation of total- and allele-specific read counts, integer copy number calls and clonal-frequency related cellular fraction (cf), corrected for tumor purity, ploidy and clonal heterogeneity. With a mean tumor purity of 80% for samples, those regions with a copy number threshold> 2.25 for trisomies, <1.25 for monosomies, and a minimum distance of 500kb were selected for CNV analysis **(Additional file 3)**. The copy-number variation genome fraction (%) *per tissue sample* was calculated as the summary of the extension of CNV-affected regions over the extension of the total genome (3.10^9bp) (**Additional file 5B**).

The SNP and CNV identified in all samples were collapsed and the distance matrix was obtained using the Manhattan estimator [1]. Then, the Neighbor-Joining Tree Estimation using the ape R

library [2] was applied. The visualization of the tree was used iTol web server [3] (**Additional file 5C**).

Functional analysis of WES data from patient#009

To address the genetic landscape of patient#009 throughout tumor transformation and progression, common and proper DNA alterations from each sample affecting genes and pathways related to cancer were grouped for further analyses (**Figure 1B**):

*****_Common germline alterations_, including germline alterations present in all tissue samples (G∩A∩B∩C∩D);

*_Common somatic alterations_, including alterations common to all tissue samples (A∩B∩C∩D);

*_Adjacent nevus alterations_, including alterations present in the nevus adjacent to CM, not present in the nevus distal from CM ((B!) U (B∩C) U (B∩D) U (B∩C∩D));

*_CM alterations_, including common alterations to primary-CM and LN-mts ((C!) U (C∩D));

*_LN-mts alterations_, including proper alterations of LN metastasis (D!).

For functional data analysis, genes with SNP/INDEL and CNV alterations at each step of progression were grouped; and a list of genes associated with KEGG pathways and cancer hallmarks [13] was filtered in our data **(Additional file 4)**. The distribution of SNP-allele frequencies and CNV-cellular fractions were analyzed through statistical tests (Wilcoxon and one-way ANOVA tests; $p<0.05$ as significant threshold) and visualized with GraphPad Prism 8.0. Cancer-driver genes were characterized by the COSMIC resource (v94) with the Cancer Gene Census catalog; a selection of driver genes was depicted in an oncoprint **(Figure 2)** [14]. Functional enrichment through KEGG, GO and Reactome resources were performed and represented using the method described in Wu T *et al.*; a p-value adjusted by FDR of 0.05 has been selected as a significant threshold **(Figure 3A**, **Additional file 5)** [15]. A distance matrix of SNP/CNV alterations from the different groups related to each step of progression was performed as described (**Figure 3B**).

REFERENCES

1. Gershenwald JE, Scolyer RA. Melanoma Staging: American Joint Committee on Cancer (AJCC) 8th Edition and Beyond. Ann Surg Oncol. 2018;25:2105–10.

2. Ladstein RG, Bachmann IM, Straume O, Akslen LA. Ki-67 expression is superior to mitotic count and novel proliferation markers PHH3, MCM4 and mitosin as a prognostic factor in thick cutaneous melanoma. BMC Cancer. 2010;10:140.

3. Mordoh J, Pampena MB, Aris M, Blanco PA, Lombardo M, von Euw EM, et al. Phase II Study of Adjuvant Immunotherapy with the CSF-470 Vaccine Plus Bacillus Calmette–Guerin Plus Recombinant Human Granulocyte Macrophage-Colony Stimulating Factor vs Medium-Dose Interferon Alpha 2B in Stages IIB, IIC, and III Cutaneous Melanoma Patie. Front Immunol. 2017;8:625.

4. Li H, Durbin R. Fast and accurate long-read alignment with Burrows–Wheeler transform. Bioinformatics. 2010;26:589–95.

5. McKenna A, Hanna M, Banks E, Sivachenko A, Cibulskis K, Kernytsky A, et al. The genome analysis toolkit: A MapReduce framework for analyzing next-generation DNA sequencing data. Genome Res. 2010;20:1297–303.

6. Law MH, Bishop DT, Lee J, Brossard M, Martin N, Moses E, et al. Genome-wide meta-analysis identifies five new susceptibility loci for cutaneous malignant melanoma. Nat Genet. 2015;47:987–95.

7. Bhandaru M, Martinka M, Li G, Rotte A. Loss of XRCC1 confers a metastatic phenotype to melanoma cells and is associated with poor survival in patients with melanoma. Pigment Cell Melanoma Res. 2014;27:366–375.

8. Rendleman J, Shang S, Dominianni C, Shields JF, Scanlon P, Adaniel C, et al. Melanoma risk loci as determinants of melanoma recurrence and survival. J Transl Med. 2013;11:279.

9. Li C, Wang M, Wang L-E, Amos CI, Zhu D, Lee JE, et al. Polymorphisms of Nucleotide Excision Repair Genes Predict Melanoma Survival. J Invest Dermatol. 2013;133:1813–21.

10. Masica DL, Douville C, Tokheim C, Bhattacharya R, Kim RG, Moad K, et al. CRAVAT 4: Cancer-related analysis of variants toolkit. Cancer Res. 2017;77.

11. McLaren W, Gil L, Hunt SE, Riat HS, Ritchie GRS, Thormann A, et al. The Ensembl Variant Effect Predictor. Genome Biol. 2016;17:122.

12. Shen R, Seshan VE. FACETS: Allele-specific copy number and clonal heterogeneity analysis tool for high-throughput DNA sequencing. Nucleic Acids Res. 2016;44:1–9.

13. Zhang D, Huo D, Xie H, Wu L, Zhang J, Liu L, et al. CHG: A Systematically Integrated Database of Cancer Hallmark Genes. Front Genet. 2020;11.

14. Gu Z, Eils R, Schlesner M. Complex heatmaps reveal patterns and correlations in multidimensional genomic data. Bioinformatics. 2016;32.

15. Wu T, Hu E, Xu S, Chen M, Guo P, Dai Z, et al. clusterProfiler 4.0: A universal enrichment tool for interpreting omics data. Innov (New York, NY). 2021;2.
