# Supplementary material for: Early contribution of germline and nevi genetic alterations to a rapidly-progressing cutaneous melanoma patient: a case report
Source: BMC Med Genomics. 2023 Jan 5;16:1. doi: 10.1186/s12920-022-01426-2 (PMC9814418; doi:10.1186/s12920-022-01426-2)
Supplement: Supplementary file 5 — Additional file 5. Characterization of somatic alterations from patient#009. [file 12920_2022_1426_MOESM5_ESM.pdf]

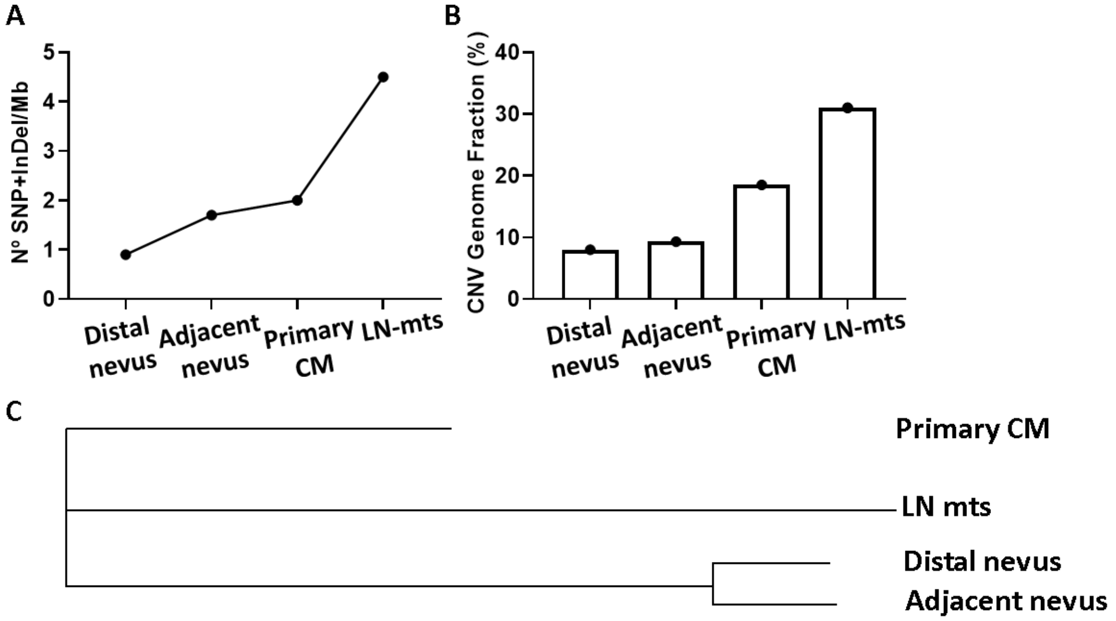

Additional file 5. Characterization of somatic alterations from patient#009. (A) Tumor mutational burden (TMB) of tissue samples, calculated as the total number of SNP plus Indels *per megabase*. **(B)** Copy-number variation genome fraction (%) *per tissue sample*, calculated as the extension of CNV regions over the extension of the genome. **(C)** Tree visualization of phylogenetic distances between all tissue samples. All analyses performed are fully described under Methods **(Additional file 1)**.
