# Supplementary material for: Early contribution of germline and nevi genetic alterations to a rapidly-progressing cutaneous melanoma patient: a case report
Source: BMC Med Genomics. 2023 Jan 5;16:1. doi: 10.1186/s12920-022-01426-2 (PMC9814418; doi:10.1186/s12920-022-01426-2)
Supplement: Supplementary file 6 — Additional file 6. Frequency distribution of alterations in patient#009 throughout progression. [file 12920_2022_1426_MOESM6_ESM.pdf]

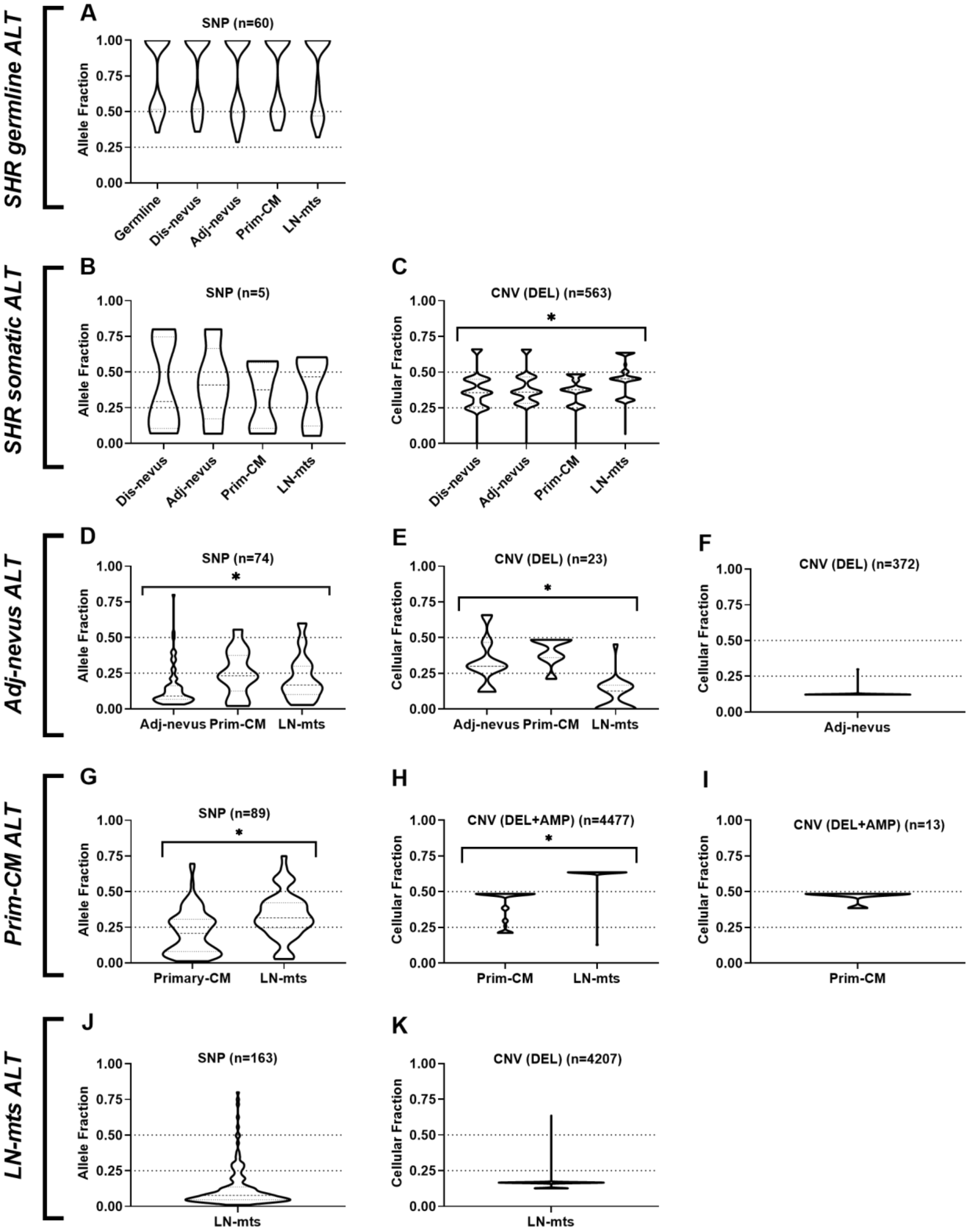

Additional file 6. Frequency distribution of alterations in patient#009 throughout progression. Distribution of SNP-allele fractions **(A, B, D, G, J)** and CNV-cellular fractions **(C, E, F, H, I, K)** abundancies throughout each step of progression. Differential distribution of SNP-allele fractions and CNV-cellular fractions were analyzed through statistical tests (Wilcoxon and one-way ANOVA), with $p<0.05$ as a significant threshold. *Subsets:* shared germline alterations (*SHR germline ALT*), shared somatic alterations (*SHR somatic ALT*), adjacent nevus alterations (*Adj-nevus ALT*), primary-CM alterations (*Prim-CM ALT*), Lymph-Node metastasis alteration (*LN-mts ALT*). *Alterations:* Single Nucleotide Polymorphisms and Insertions–Deletions (SNP/INDEL); Copy Number Variations included copy-number deleted (DEL) and amplified (AMP) genes. All analyses performed are fully described under Methods **(Additional file 1)**.
