# Supplementary material for: Early contribution of germline and nevi genetic alterations to a rapidly-progressing cutaneous melanoma patient: a case report
Source: BMC Med Genomics. 2023 Jan 5;16:1. doi: 10.1186/s12920-022-01426-2 (PMC9814418; doi:10.1186/s12920-022-01426-2)
Supplement: Supplementary file 7 — Additional file 7. Complementary functional analysis of altered genes in Patient#009. [file 12920_2022_1426_MOESM7_ESM.pdf]

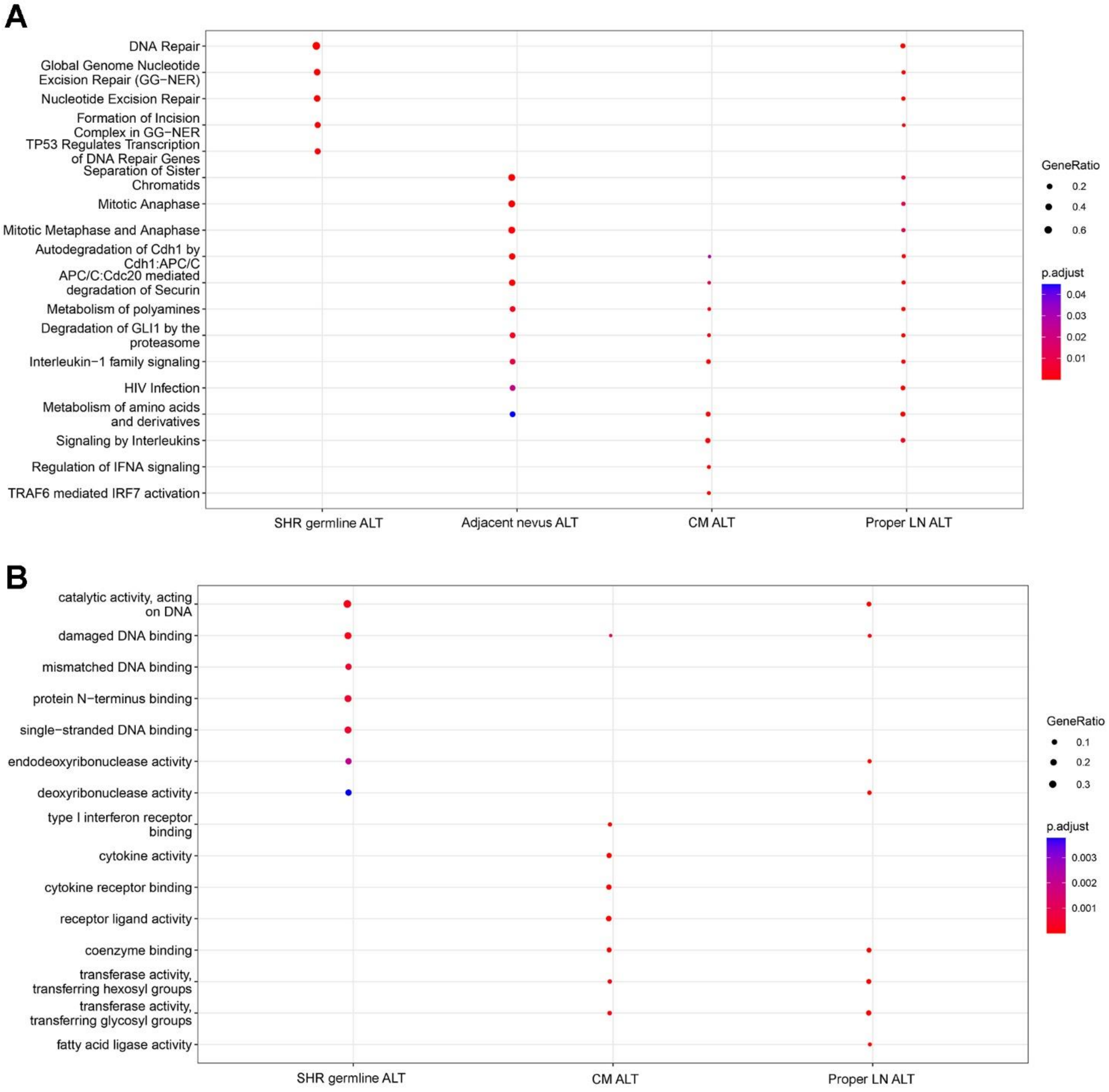

Additional file 7. Complementary functional analysis of altered genes in Patient#009. Functional enrichment as determined by Reactome **(A)** and GO **(B)** resources of selected SNP/CNV alterations at each step of progression.
